# The Role of Cytokines in Traumatic Brain Injury

**DOI:** 10.3390/biomedicines14040879

**Published:** 2026-04-12

**Authors:** Lamprini Vlachodimitropoulou, Marios Lampros, George A. Alexiou, Anastasia K. Zikou, Spyridon Voulgaris, Paraskevi V. Voulgari

**Affiliations:** 1 Department of Neurosurgery, School of Medicine, University of Ioannina, 45500 Ioannina, Greece; 2 Department of Radiology, School of Medicine, University of Ioannina, 45500 Ioannina, Greece; 3 Department of Rheumatology, School of Medicine, University of Ioannina, 45500 Ioannina, Greece; pvoulgar@uoi.gr

**Keywords:** traumatic brain injury, cytokines, prognosis, outcome, neuroinlfammation

## Abstract

Traumatic brain injury (TBI) is a major cause of death and disability, mainly in persons under 45 years of age and it remains clinically challenging due to its heterogeneous pathophysiology and unpredictable course. Except from the initial mechanical damage, secondary injury —largely driven by neuroinflammation—plays a critical role in outcome and extent of recovery. Cytokines are central mediators of this immune response and have therefore been extensively studied as potential biomarkers for TBI diagnosis, need of imaging and prognosis. Among pro-inflammatory cytokines, IL-1β is rapidly upregulated after TBI and contributes to blood–brain barrier disruption and secondary damage. Furthermore, experimental studies suggest that IL-1 inhibition could be neuroprotective. IL-6 is up to date the most extensively studied cytokine and shows strong associations with injury severity, neuroimaging abnormalities, mortality and long-term functional outcomes across multiple adult and pediatric studies. Nevertheless, results vary depending on the biological compartment and timing. Anti-inflammatory IL-10 levels correlate with injury severity and has shown promise in distinguishing CT-positive from CT-negative mild TBI patients, potentially reducing unnecessary imaging, though findings are inconsistent. Other cytokines, including IL-17, TNF-α, IL-8, IL-9, and IL-15, have been correlated to post-traumatic neuroinflammation and may have diagnostic or prognostic value. Overall, IL-6 and IL-10 currently appear to be the most promising cytokine as biomarkers, however future research should focus on standardized cytokines assessment methods and possible use of multimarker panels.

## 1. Introduction

Traumatic brain injury (TBI) is a leading cause of death and disability for people under 45, with significant economic and societal consequences. Approximately 50% of people worldwide will suffer a TBI at some point in their lives [1]. The majority of TBIs are mild, followed by severe and moderate cases. Even with recent improvements in our understanding of the pathophysiology of brain injury, it is still very difficult to accurately predict prognosis. The diverse nature of TBI, which includes a dynamic interaction between mechanical damage, subsequent metabolic changes, and dysregulation of the immune response, play a pivotal role on the clinical complexity [2,3].

Both secondary injury and long-term recovery are significantly influenced by the prolonged and complex immune response that is triggered by TBI. CD4 T cells can be both neuroprotective and neurotoxic and are important regulators of neuroinflammatory processes among adaptive immune cells. Cytokines are polypeptides that are acutely increased in response to pathological or stressful conditions and mediate the inflammatory cascade that is triggered by TBI [4,5]. Apart from their role in the immune system, cytokines are essential molecules for maintaining tissue homeostasis and constitute mediators of intracellular communication. Examples of pro-inflammatory cytokines are tumor necrosis factor-alpha (TNF-α), interleukin-1 β (IL-1β), and interleukin-6 (IL-6). These cytokines are increased after brain injury and contribute to subsequent tissue damage [4,5,6]. Conversely, anti-inflammatory cytokines, such as interleukin-10 (IL-10), have a protective role, since they promote tissue repair and reduce inflammation [7] [Table 1 and Table 2].

Neuroinflammation has emerged as an important therapeutic target in the treatment of TBI due to its critical role in the development of secondary injury. Reactive oxygen species (ROS) produced by NADPH oxidase (NOX) function as essential signaling molecules that increase the synthesis of pro-inflammatory cytokines, resulting in a self-sustaining loop of neuroinflammation and injury [8]. In the acute phase injured cells also release damage-3associated molecular patterns (DAMPs), which trigger a cytokine storm and proinflammatory microglia activation [9]. NOX inhibitors block the production of ROS by targeting NADPH oxidase enzyme complexes. In the context of epilepsy, early NOX2 inhibition successfully lowered oxidative stress, neuronal damage and seizure burden [10].

Several biomarkers in blood, cerebrospinal fluid, saliva, tears, or urine have been investigated in patients with TBI. Biomarkers may have diagnostic, prognostic, and monitoring roles in TBI. Their role in discriminating patients with mild TBI in need of neuroimaging, for detecting patients that will develop coagulopathy, for prognostic purposes and for monitoring therapy response is under investigation [11]. To date, only a few of these biomarkers have received clinical approval, such as S100 calcium-binding protein B in the Scandinavian neurotrauma guidelines [12] and a combination panel of Glial Fibrillary Acidic Protein (GFAP) and Ubiquitin Carboxy-terminal Hydrolase L1 (UCH-L1) by the Federal Drug Authority (FDA) [13], however, only for identifying patients with mild TBI in need of CT. The present study is a narrative review of the role of cytokines as biomarkers in patients with TBI. The selection of cytokines studied was based on their well-established involvement in neuroinflammatory pathways after TBI and the availability of evidence linking them to clinical outcomes, injury severity and prediction of the need for CT.

## 2. IL-1

There have been prior reports of an inflammatory response in the brain following TBI, which may be strongly associated with a number of pro-inflammatory cytokines [14]. Among these, activated microglia, astrocytes, and endothelial cells in the brain generate the pro-inflammatory cytokine IL-1β, which functions by attaching to its cell surface receptor IL-IR [15]. Neural plasticity, immune cell activation, and blood–brain barrier permeability are all impacted by IL-1. As early as one hour after the injury, elevated levels of IL-1β mRNA and protein are seen in the cortex and deep central brain regions in both mild and severe TBI. It has been demonstrated that neutralizing IL-1β with a monoclonal antibody can stop secondary damage by preventing downstream microglial activation in a mouse model [15]. Furthermore, the interleukin-1 receptor antagonist (IL-1ra) treatment is likely to have an impact. An endogenous antagonist to IL-1 is also available in recombinant form and has been proven to be safe in TBI patients [16]. A clinical trial is currently underway to investigate its efficacy in moderate-to-severe TBI patients. Neuroinflammatory mechanisms linked to neurodegeneration are influenced by this fast IL-1β production [17]. Nevertheless, most evidence remains experimental or tested in few patients and thus should be interpreted with caution. Regarding clinical trials, in the prospective TRACK-TBI Pilot (Transforming Research and Clinical Knowledge in Traumatic Brain Injury Pilot) study IL-1b could discriminate TBI patients from orthopedic controls (AUC = 0.795) and patients with positive CT versus patients with negative CT (0.757) findings. The AUC was <0.7 for predicting 3- and 6-month Glasgow Outcome Scale-Extended (GOSE) outcome [18].

## 3. IL-6

The multifunctional cytokine IL-6 is involved in tissue healing, immunological activation, inflammation, and metabolic control. Microglia, astrocytes, neurons, endothelial cells, and invading immune cells all generate it in the central nervous system (CNS) following TBI. IL-6 is involved in neuroinflammation, blood–brain barrier disruption, and secondary injury processes and is rapidly upregulated following tissue injury [19]. In the prospective TRACK-TBI Pilot patients with TBI were triaged for head CT and had blood samples taken within 24 h of the injury. There were orthopedic controls and healthy controls. From plasma, thirty-one inflammatory markers were examined. IL-6 could discriminate moderate/severe from mild TBI (AUC = 0.747) and patients with positive CT versus patients with negative CT (0.757). The AUC was <0.7 for predicting 3- and 6-month GOSE outcome [18]. Concentrations of IL-6 were also found elevated in patients with positive CT, as well as positive MRI compared to controls, even after adjusting for age, sex, and 33risk factors for cardiovascular disease (CVD), such as hypertension and hyperlipidemia. Proinflammatory cytokines may be increased in patients with CVD; however, based on the results so far, IL-6 may have clinical utility even in these patients [20]. For distinguishing military combat personnel with mild TBI from those without, acute IL-6 levels demons3trated a good predictive value (AUC 0.82 [95% CI 0.72–0.90]) [21]. Comparable outcomes were observed when comparing athletes with and without mild TBI [22].

In a well-designed prospective study the ability of serum IL-6 to predict mortality and disability in patients with moderate and severe TBI was investigated. Admission GCS, age, Rotterdam score, hospital infections, and day-0 IL-6 were clinical factors linked to the 6-month unfavorable outcome. Day-0 IL-6 was substantially linked to the unfavorable result at six months after controlling for age, the extent of the injury, and the existence of a hospital infection. At a cut-off value of 59 pg/mL, the calculated sensitivity and specificity were 75% and 89%, respectively [23].

In a retrospective study that included 122 patients with moderate-to-severe TBI, the GOS scores at 6 months after discharge were recorded. IL-6 levels were an independent predictor of outcome. Prognostic performance analysis demonstrated that IL-6 achieved an AUC of 0.716 [24]. Similar results were reported in a study that was performed on 109 adult patients with TBI, 20 adult healthy controls and a group of 17 pediatric patients. Elevated levels of IL-6 were detected on day 1 post-trauma; higher levels of IL-6 were associated with more severe TBI and more severe brain imaging findings. On a multivariate logistic regression analysis only IL-6 at day-1 was an independent predictor of an unfavorable outcome [25]. IL-6 levels at day one have also been associated with more severe brain imaging findings [26].

A few years ago, Ooi et al. conducted a systematic review investigating the role of IL-6 as a prognostic biomarker [27]. Studies with adult TBI patients whose blood, cerebrospinal fluid, and/or brain parenchyma IL-6 concentrations were examined in relation to functional outcome and/or mortality were included. There were fifteen trials totaling 699 patients. The pooled mean age was 40.8 years, the majority of patients (71.7%) were male, and 78.1% had severe TBI. IL-6 levels were determined in serum in eleven trials, cerebrospinal fluid in six, and parenchyma in one. Higher IL-6 concentrations were linked to worse outcomes in five serum trials, whereas there was no significant correlation in the other five. Higher IL-6 levels were linked to worse outcomes in one CSF study, better outcomes were predicted in another, and no association was seen in three. Better results were linked to higher levels of parenchymal IL-6 [27].

## 4. IL-10

One important anti-inflammatory cytokine that is essential for controlling immunological responses is interleukin-10 (IL-10). It is produced by a variety of cell types, such as macrophages, T lymphocytes, and microglia, and it mainly limits immune-mediated tissue damage and inhibits the synthesis of pro-inflammatory cytokines. By lowering secondary damage processes, IL-10 may have neuroprotective benefits in the context of central nervous system injury by modulating neuroinflammation. In adult patients with TBI increased levels of IL-10 have been reported on day 1 [26]. Furthermore, higher levels of IL-10 were reported to be associated with more severe TBI according to widely used clinical and functional scales [26].

An important issue in mild TBI is the discrimination of patients with negative CT from patients with positive CT. Although most patients with mild TBI have normal neuroimaging, a clinically significant percentage have positive results on head CT. Depending on patient age, the mechanism of injury, and clinical risk factors, reported incidence rates of CT abnormalities in mild TBI typically fall between 5 and 15%. Small intracranial hemorrhages, cerebral contusions, and skull fractures are common findings; the majority of these do not necessitate neurosurgical intervention. Finding positive CT results is still crucial because it directs acute care, surveillance techniques, and risk assessment for neurological decline [28]. More importantly, according to a 2025 study published in JAMA Internal Medicine, ionizing radiation from current CT scan practices in the United States could result in approximately 103,000 cancer cases per year, which could account for 5% of new diagnoses. The majority of these cases occur in adults due to high scan volumes, but children are more at risk. The study emphasized dose reduction and justification to balance benefits and risks [29]. Khosh-Fetrat et al. investigated whether IL-10 could distinguish CT-positive from negative patients with mild TBI in a prospective observational setting. Measurements were performed in blood less than three hours after hospital admission using ELISA in a sample of 300 patients. The group with positive CT scans had considerably higher levels of IL-10 (*p* < 0.001). The specificity of IL-10 was only 38.1% when the sensitivity was set to 100%. Classification performance considerably improved at the 100% sensitivity level with a 93% specificity for mild TBI patients aged 36 to 66 [30]. Nevertheless, Koivikko et al. after analyzing IL-10 levels in 184 patients with TBI and 39 controls found that IL-10 could not discriminate CT-positive or CT-negative patients with mild TBI from controls. Patients with mild TBI discharged from the emergency department exhibited lower levels of IL-10 in comparison to those admitted to the neurosurgical ward [31].

## 5. IL-17

After TBI, interleukin-17 (IL-17), a pro-inflammatory cytokine, is crucial to the secondary damage cascade. Th17 cells, γδ T cells, and innate immune cells are the main producers of IL-17, which disrupts the blood–brain barrier, attracts neutrophils, and increases neuroinflammation. Increased production of other inflammatory mediators, increased glial activation, and neuronal damage have all been linked to elevated IL-17 levels following TBI. IL-17 is additionally a possible therapeutic target in TBI, as experimental research indicates that blocking IL-17 signaling can reduce neuroinflammation and enhance neurological outcomes [32]. Even if the results are encouraging, the majority of the data comes from experimental research in animal models. Translational limitations exist and applicability in humans requires further research. In a study that included 140 active-duty, injured service members (59 with TBI and 81 non-TBI), only IL-17A was the only biomarker elevated significantly in both serum and effluent in TBI compared to non-TBI casualties [33].

## 6. TNF-α

TNF-α plays a complex role in TBI, depending on a number of variables, including the local microenvironment, the severity of the injury, and timing. In a variety of diseases, TNF-α demonstrates a range of biological roles as a ligand and receptor. TNF-α causes cytotoxic effects as a ligand. TNF-α can control T-cell functions as a receptor. The precise mechanism of TNF-α in TBI is multifaceted, supporting neurotrophic and healing processes in addition to having pro-inflammatory and neurotoxic effects [34]. Although TNF-α can cause an anti-inflammatory response, there is currently little research on it as a therapeutic target for TBI, mainly in animal studies. In a small prospective study of patients with isolated TBI when compared to the control group, TNF-α concentrations were significantly higher in the TBI group. Higher blood levels of TNF-α, however, did not significantly correlate with a fatal outcome. Measurements were performed after a mean time of 10.2 h after injury [35]. Similar to the proinflammatory IL-6, in a study of 250 patients with mild TBI, TNF-α was elevated in patients with positive CT and MRI findings compared to controls. Furthermore, TNF-α could differentiate patients with findings on CT compared to controls [20].

## 7. Other Cytokines

IL-8 is a pro-inflammatory chemokine that is produced by microglia, astrocytes, endothelial cells, and infiltrating immune cells after TBI. An increase in IL-8 post-injury has been reported, although a study found reduced levels in TBI patients compared to controls [25]. One well known consequence of TBI is post-traumatic vasospasm. Vasospasm is frequently linked to traumatic subarachnoid hemorrhage and typically appears a few days after the injury. Although the precise mechanism is unclear, blood breakdown products and inflammation are thought to be crucial. Secondary brain damage and a deterioration of neurological status are possible outcomes of vasospasm. Low levels of IL-9 were reported as a predictor of vasospasm development [36]. The pro-inflammatory cytokine interleukin-15 (IL-15) is crucial to both innate and adaptive immune responses. It is produced by monocytes, macrophages, and endothelial cells and contributes to the activation and growth of memory T lymphocytes and natural killer (NK) cells. IL-15 has been linked to immune cell recruitment, persistent neuroinflammation, and possible subsequent tissue damage in the context of CNS injury. In the prospective TRACK-TBI Pilot study IL-15 could discriminate moderate/severe from mild TBI (AUC = 0.720) and patients with positive CT versus patients with negative CT (0.724). At 3 months, IL-15 (AUC = 0.738) discriminated GOSE 5–8 from 1–4. At 6 months, IL-15 discriminated GOSE 1–4 from 5–8 (AUC = 0.704) and GOSE <8/= 8 (0.711) [18].

## 8. Pediatric TBI

The incidence of pediatric TBI varies widely by country and area worldwide. The estimated global incidence of pediatric TBI has been reported to be 226.4 cases per 100,000 children yearly. TBI is the cause of 1.9 deaths per 100,000 children annually. Regarding severity, 11.0% of children experience severe TBI. The overall mortality rate has been reported to be 1.4% [37]. Data from the National Center for Health Statistics in the USA showed that 6.8% of children under the age of 17 reported having experienced symptoms of a brain injury or concussion [38]. The age distribution of TBI in children is bimodal, with the majority of injured children being younger than two or older than fifteen. Identifying reliable TBI biomarkers in children is of paramount importance. First, in mild TBI exposure to radiation via CT imaging has been associated with increased lifetime incidence of cancer [39]. Furthermore, serial imaging or invasive intracranial pressure measurement in children is more difficult to perform compared to adults. Cytokines have been studied in children for diagnostic, prognostic, and monitoring purposes [40].

In a recent study flow cytometry was used to measure the levels of plasma IL-1β, IL-2, IL-4, IL-5, IL-6, IL-8, IL-10, IL-12p70, IL-17A, IFN-α, IFN-γ, and TNF-α in 50 children with moderate and severe TBI and 20 controls. Measurements were performed at admission and on days 5 to 7. The baseline levels of IL-6 and IL-8 were significantly higher in non-survivors than in survivors. Additionally, children with adverse outcomes had significantly higher baseline levels of IL-5, IL-6, and IL-8. In comparison to their respective baselines, the levels of IL-6 and IL-8 were considerably lower in survivors and higher in non-survivors on days 5 to 7. Baseline levels of IL-1β, IL-2, IL-4, IL-10, IL-12p70, IL-17A, IFN-γ, IFN-α, and TNF-α were not linked to an adverse 6-month outcome or in-hospital mortality [25]. In a study that included 104 children with mild TBI, 6 with severe TBI and 98 controls there was elevated IL-6 in mild TBI patients relative to controls, reduced levels of TNF-α, IL-8, IL-10, and IL-17A in mild TBI relative to controls, considerably elevated IFN-γ in mild TBI patients relative to controls, whereas IFN-γ was considerably lower in patients with severe TBI than in controls [41].

In CSF IL-6 levels rise dramatically after TBI, whereas IL-1β levels gradually increase over the first two hours following injury and then continue to rise for the next 48 h [42]. Severe head injuries and poor neurological outcomes are linked to an early rise in IL-1β levels [43]. In a prospective cohort study IL-6 and vascular homeostatic (angiopoietin-2 [AP-2], endothelin-1 [ET-1], endocan-2 [EC-2]) were evaluated in the blood of children with TBI. A relationship between inflammation and alterations in vascular homeostasis was found [44].

## 9. Genetic Polymorphisms

Rehabilitation and prognosis may be impacted by genetic variations in cytokine-coding genes that regulate cytokine expression. In a study that evaluated, in 28 subacute TBI patients, the genetic polymorphisms in the TNF-α, IL-6, IL-6 receptor, IL-1β, and IL-10 genes there was a correlation between lower GOSE scores and the IL-6-174 (GG) and IL-6R 1073 (AA) genotypes (*p* = 0.02 and *p* = 0.01, respectively). Poorer results were associated with co-segregation of IL-6-174 and IL-6R 1073 G-A alleles (*p* = 0.01). Following rehabilitation, patients with the TNF-α-308 (GA) genotype had a greater incidence of post-traumatic confusional state (*p* = 0.03) and showed less recovery in Barthel and Mobility ratings (*p* = 0.001 and *p* = 0.01, respectively). All things considered, the TNF-α-308 (GA), IL-6-174 (GG), and IL-6R 1073 (AA) genotypes have a detrimental effect on rehabilitation outcomes, perhaps because they increase neuroinflammation [45]. Nevertheless, the results so far from other studies are ambiguous. For instance, the IL-6-174 (GG) genotype has been previously associated with positive impact on TBI outcomes [4], while others have found no link between them [5]. However, other researchers have reported worse results in patients with the same genotype [6]. The dual function of IL-6, which can operate as a pro-inflammatory cytokine that increases cerebral edema but may also promote neurogenesis and shift microglia toward an anti-inflammatory phenotype, may account for these disparities.

## 10. Conclusions

Several studies have been performed to explore the multiple roles of cytokines in TBI. The vast majority of studies have been focused on the prediction of patients’ outcomes. The reason is that current clinical tools are limited in their ability to predict patients’ outcomes following severe TBI. There are few studies investigating the role of cytokines in predicting the need for CT in mild TBI patients. Other applications that could be investigated in the future are monitoring TBI progression and evaluating treatment response. Furthermore, there are several limitations of the current studies. Many studies have a limited number of patients, different methods to assess cytokine concentrations, measurements at different time points that affect cytokine concentrations and there is a lack of cerebrospinal fluid cytokine level measurements, which would have offered a more thorough mechanistic understanding of the noted correlations. Finally, TBI severity is a crucial factor that influences cytokine concentration and several studies are focused on different TBI severities. IL-6 and IL-10 are among the most extensively studied cytokines in TBI and may have potential as biomarkers, although findings across studies remain inconsistent. Based on the results so far, cytokines may be better suited as part of a multiple biomarker panel including markers with different TBI pathophysiological mechanisms.

## Figures and Tables

**Table 1:** Summary of cytokines investigated as biomarkers in TBI.

Cytokine	Main Cellular Sources	Biological Role	Clinical Relevance
IL-1β	Microglia, astrocytes, endothelial cells	Potent pro-inflammatory mediator; BBB disruption; microglial activation	Discriminates TBI vs. controls and CT+ vs. CT−; limited prognostic value for long-term outcome
IL-6	Microglia, astrocytes, neurons, endothelial cells	Inflammation, BBB disruption, metabolic regulation	Strong association with injury severity, CT/MRI positivity, mortality, and functional outcome
IL-10	Macrophages, T lymphocytes, microglia	Anti-inflammatory; limits immune-mediated damage	Reflects injury severity; potential tool to exclude CT lesions in selected mTBI populations
IL-17A	Th17 cells, γδ T cells, innate immune cells	Neutrophil recruitment; BBB disruption	Elevated in serum and effluent in TBI; possible therapeutic target
TNF-α	Microglia, macrophages, neurons	Dual role: pro-inflammatory and neurotrophic	Elevated in TBI and CT+ cases; inconsistent association with outcome
IL-8	Microglia, astrocytes, endothelial cells	Chemotaxis, inflammation	Altered levels reported; possible association with pediatric outcomes
IL-9	T cells	Immune modulation	Low levels associated with post-traumatic vasospasm
IL-15	Monocytes, macrophages, endothelial cells	T-cell and NK-cell activation	Discriminates TBI severity, CT status, and functional outcome

**Table 2:** Kinetic profiles and temporal upregulation of cytokines in TBI.

Phase	Timeframe	Biomarker(s)	Kinetic Description and Duration
Hyper-Acute	<1 h	IL-1β	Rapidly upregulated mRNA and protein levels in the cortex and deep brain regions.
Acute	3–24 h	IL-6, IL-10, TNF-α	IL-6: Rapid upregulation following tissue injury, staying elevated through the first 24 h. IL-10: Increased levels reported specifically on day 1. TNF-α: Significant elevation measured at a mean of 10.2 h post-injury.
Sub-Acute	Days 1–7	IL-8, IL-15, IL-17A	IL-8: Baseline elevation at admission; levels remain significantly higher in non-survivors through days 5 to 7. IL-17A: Sustained elevation in both serum and effluent.
Delayed	Weeks	IL-9	Characterized by a later immune modulation profile; low levels are linked to post-traumatic vasospasm which typically appears days after the initial injury.

## Data Availability

No new data were created or analyzed in this study. Data sharing is not applicable to this article.
